# Molecular epidemiology of hepatitis B virus infection in Norway

**DOI:** 10.1186/s12879-019-3868-8

**Published:** 2019-03-07

**Authors:** John H.-O. Pettersson, Solveig Myking, Hilde Elshaug, Kirsten Irene Ege Bygdås, Kathrine Stene-Johansen

**Affiliations:** 1 Department of Infectious Disease Epidemiology and Modelling, Norwegian Institute of Public Health, Oslo, Norway; 2 Zoonosis Science Center, Department of Medical Biochemistry and Microbiology, Uppsala University, Uppsala, Sweden; 3 Marie Bashir Institute for Infectious Diseases and Biosecurity, Charles Perkins Centre, School of Life and Environmental Sciences and Sydney Medical School, the University of Sydney, Sydney, NSW 2006 Australia; 4 Public Health Agency of Sweden, Nobels väg 18, SE-171 82 Solna, Sweden; 5 Department of Infectious Disease Registries, Norwegian Institute of Public Health, Oslo, Norway; 6 Department of Molecular Biology, Norwegian Institute of Public Health, Oslo, Norway; 7 Department of Virology, Norwegian Institute of Public Health, Oslo, Norway

**Keywords:** Hepatitis B virus, Molecular epidemiology, Phylogenetics, Genotyping

## Abstract

**Background:**

Hepatitis B virus (HBV) infection remains a serious global health challenge. The widespread distribution of HBV is highlighted by multiple HBV genotypes associated with different geographical origin and transmission patterns, as well as, clinical outcomes. Investigating population HBV genotype composition and origin is therefore highly warranted.

**Methods:**

In this molecular epidemiological study we analysed 1157 HBV S-gene sequences collected from patients in Norway, primarily in the period 2004–2011, and linked them to epidemiological data from the Norwegian surveillance system for communicable diseases.

**Results:**

Of the patients with reported country of infection (*n* = 909), 10% (*n* = 93) were infected in Norway, but the majority (*n* = 816; 90%) stated that they became infected outside of Norway. Of the patients infected outside of Norway, most became infected in Southeast and East Asia (*n* = 465; 51%) and Central, West, and North Africa (*n* = 254; 28%). The distribution of HBV genotypes in Norway is dominated by genotype D (32%) followed by genotype A (22%), B and C (18 and 18%, respectively), and E (7%). Genotype B, C and E were phylogenetically categorized by a majority of sequences originating from distinct geographical regions, either Asia or Africa, whereas genotype A and D originated from multiple geographic regions. However, within genotype A and D, our molecular epidemiology analysis indicated a geographical clustering of sequences depending on their geographical origin.

**Conclusions:**

The majority of HBV patients in Norway became infected outside of Norway and were represented by most common genotypes. Patients stated to have been infected in Norway were found primarily within genotype A and D, and were phylogenetically characterized by both small local clusters and interspersed sequences that clustered with non-Norwegian sequences, indicating that a proportion of the patients assumed to have been infected in Norway likely became infected outside of Norway although assumed the contrary.

**Electronic supplementary material:**

The online version of this article (10.1186/s12879-019-3868-8) contains supplementary material, which is available to authorized users.

## Background

Viral hepatitis and hepatitis B virus (HBV) infection in particular is one of the world’s most significant public health problems [1]. Globally, ca. 260 million people are estimated to be chronically infected with HBV and annually there are close to 900,000 HBV related deaths, mainly due to cirrhosis or hepatocellular carcinoma [2–6]. Since the introduction of an effective vaccine in 1982, the global immunisation coverage of infants has gradually increased to 87% in 2016 and hence the number of new chronic infections has dramatically decreased among immunised children [5, 7].

However, the global prevalence of HBV, indicated by the proportion of chronic HBV carriers in the population that is seropositive for the hepatitis B surface antigen (HBsAg), varies strongly between different geographical regions. Broadly viewed, high endemic regions (≥ 8%) include the majority of Africa, parts of Asia, and parts of the Middle East. Intermediate endemic regions (2–8%) include eastern- and southern Europe, Russia, parts of Central- and South America, and parts Africa and eastern and southeast Asia. Low endemic regions (< 2%) include Australia and New Zealand, Northern- and Western Europe, North America and the majority of Central and South America [3, 4]. As such, Norway is classified as a low endemic country for HBV infection [8]. Both acute and chronic infections have been notifiable to the Norwegian surveillance system for communicable diseases (MSIS) since 1975 and 1992, respectively. Similar to many other low endemic regions, acute HBV infections in Norway are most commonly acquired through either intravenous drug use (> 60%) or sexual contact [8]. However, the majority of people with chronic HBV infection in Norway are people from high- and intermediate endemic regions who became infected before arriving to Norway [8]. In Norway, all migrants are offered HBV screening within 3 months on arrival. However, the test is voluntary, and no data are available for the proportion of migrants who choose not to be tested. Vaccination in Norway has until recently only been recommended for defined risk groups, such as people who inject drugs (PWID), men who have sex with men (MSM), immigrants and contacts of known carriers [8]. However, from 2017, universal HBV vaccination was implemented in the child immunisation program in Norway.

The HBV is an enveloped virus in the family Hepadnaviridae with a circular and partially double-stranded DNA genome of approximately 3.2 kbp in length. The genome consists of four partially overlapping open reading frames (S, C, P and X) encoding seven proteins (preS1, preS2 and S surface antigen; precore and core protein; polymerase protein; X protein) [9]. Based on a sequence divergence of > 7.5–8% when comparing complete genomes, HBV is currently divided into at least eight phylogenetically distinct groups, referred to as genotypes A–H with a further two tentatively proposed, genotypes I and J [9–12]. Furthermore, some genotypes have been divided into sub-genotypes with > 4% sequence diversity based on comparison of whole genome sequences. These genotypes, or even sub-genotypes, have distinct or overlapping geographical distribution [9, 13, 14]. In general, genotype A is common in sub-Sahara and Western Africa, as well as Northern Europe. In Asia genotypes B and C are highly prevalent. Genotype D is distributed worldwide, but sub-genotypes show geographic patterns. The various genotypes have in several studies shown to be associated with differences in pathogenicity, transmission modes, disease progression and response to treatment [12, 14–20]. For example, genotype C is more strongly associated with development to advanced liver disease compared to all other genotypes [15, 17, 21]. HBV genotyping may therefore serve as an important clinical and epidemiological marker [20]. HBV genotyping has consequently been a part of patient management in Norway since 2004.

This is the first molecular epidemiological study of HBV in Norway where we analyse genotype distribution related to transmission routes and geographical origin of infection among patients. The aim of the current study was to better understand the molecular epidemiology of HBV in Norway by linking HBV sequences with epidemiological data from the Norwegian surveillance system for communicable diseases (MSIS). Importantly, this will help understand the origin and distribution of HBV genotype in patients in Norway.

## Methods

### Study population

Samples submitted to the national reference laboratory for hepatitis at the Norwegian Institute of Public Health in the period 1979–2011 (*n* = 1160) for HBV genotype analysis were included in the study. Further, epidemiological data from MSIS on gender (*n* = 998), probable country of infection (*n* = 909) and route of transmission (*n* = 277) was linked to HBV-genotype and sequence data. The routes of transmission used were (i) mother to child transmission (MTCT), (ii) intravenous drug use (IDU) and (iii) sexual contact. The data include both acute and chronic infections, but the majority of cases are chronic infections as these samples were analysed for HBV genotype as part of their patient management. The study was approved by the Regional committee for medical and health research ethics (REC) South East.

### HBV-genotyping

HBV DNA was extracted from 200 μl plasma or serums samples by different methods used at the reference laboratory over the years, including the QIAamp DNA mini kit (QIAgen), Affigene extraction kit (Cepheid) and Abbot sp2000 (Abbott). The elution volume was 35–100 μl depending on the extraction method used above according to these manufactures’ protocol. HBV DNA was amplified from 5 μl extract using HBV-specific primers covering the S-gene region 5′– GACCCCTGCTCGTGTTA –3` (forwards) and 5′– TGAATACTTTCCAATCAATAGG – 3′ (reverse) using AB-gene PCR-buffer with SYBR green (0.33x), Pt-Taq, 3 mM Mg, 0.5 μM primer, 0.2 mM dNTP and an annealing temperature of 65 °C. The PCR products (808 bp) were sequenced using Big Dye Terminator v1.1 on the ABI Prism 3100 instrument (Applied Biosystems). All sequences produced in the present study have been deposited in NCBI GenBank (Accession numbers MK173066–MK174222). The viral samples were genotyped by submitting sequence of the S-gene to the HBV genotyping databases at NCBI (https://www.ncbi.nlm.nih.gov/projects/genotyping/formpage.cgi) and/or at the Max-Planck-Institute for informatics (https://hbv.geno2pheno.org/). The genotyping results were also confirmed in subsequent phylogenetic analyses.

### Sequence alignment and model-testing

An HBV S-gene multiple sequence alignment was constructed with MAFFT v.7 [22] using the G-INS-i standard settings and was visualized and edited in AliView [23]. The alignment consisted of a total of 1512 S-gene HBV sequences, that included 1157 sequences sampled in Norway, in the period 1979–2011, and 355 reference sequences, sampled between 1980 and 2013, retrieved from NCBI (Additional file 1: Table S1). The NCBI sequences were chosen based on available information on year of isolation, genotype, geographical origin, as well as genetic similarity to the Norwegian sequences determined by the NCBI BLAST. The alignment was trimmed to a size of 740 bp. To select the most suitable evolutionary nucleotide substitution model, model-testing was conducted using jModelTest 2 [24]. In all subsequent analyses, a general time reversible model (GTR) of nucleotide substitution, with a proportion of invariant sites (I) and gamma distribution of rates across sites with four rate categories (G4) was used.

### Phylogenetic interference and molecular epidemiology

To place the Norwegian S-gene HBV sequences in context with those of other studies, a phylogeny was inferred based on the alignment consisting of 1157 sequences collected in Norway and 355 reference sequences from NCBI using MrBayes v.3.2.2 [25]. This was done by executing two parallel runs with four Metropolis-coupled chains for 25 million Markov chain Monte Carlo (MCMC) generations, using GTR + I + G4, sampling every 1000 generations and run with default dirichlet priors, discarding the first 25% as burn-in and then summarized as a consensus tree. The phylogenetic tree was viewed and edited in FigTree v.1.4.2 (http://tree.bio.ed.ac.uk/software/figtree/).

## Results

**Fig. 1:**
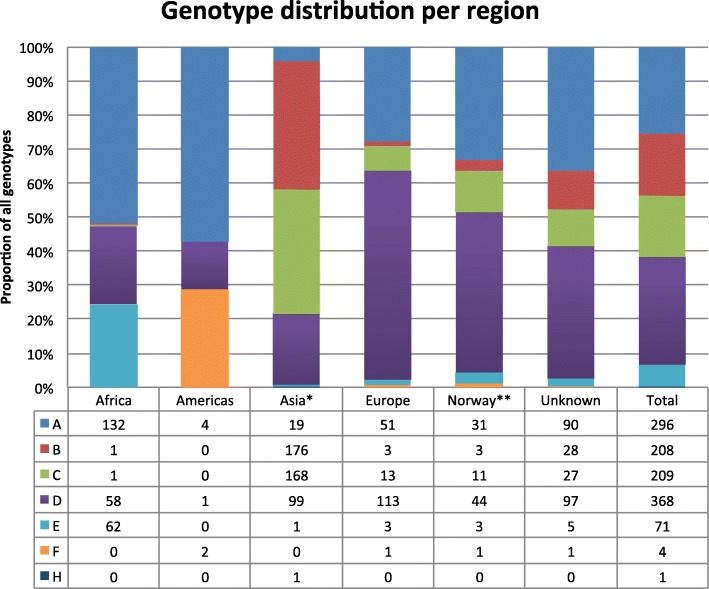
The HBV genotype distribution among patients in Norway according to the probable country of infection. The samples from patients infected in Norway are also part of the European distribution. “Unknown” are cases with no information of origin of infection or country of birth. * = includes one sample from Australia. ** = include only samples from patients stated to have become infected in Norway

After excluding three samples due to short sequence length and/or poor sequence quality, a total of 1157 samples collected from HBV infected patients in Norway between 1979 and 2011 were genotyped by sequencing the HBV S-gene. Of these samples, 93 were from patients assumed to have become infected within Norway, where as 1064 sequences were from patients assumed to be infected outside Norway (*n* = 816) or most probably, although unknown, being infected before migration to Norway (*n* = 248). In the latter case place of birth or origin was selected as origin of infection. The gender balance of males and females were 63/37 (males = 627, females = 371). The distribution of genotypes among these 1157 patients is shown in Fig. 1 according to their geographical origin of infection. Including all samples collected in Norway, the distribution of genotypes were genotype D (32%), genotype A (22%), genotype C (18%), genotype B (18%) and genotype E (6%). Genotype F and H were detected on very few occasions (*n* = 4 and *n* = 1, respectively). Among samples from people probably being infected in Africa (*n* = 254), genotype A (52%), D (23%) and E (25%) were the main genotypes. Among samples from people probably being infected in Asia (*n* = 464), genotype B (38%) and C (36%) are the predominant genotypes, but also genotype D (21%) contributes to almost a fifth of the Asian cases analysed. In persons probably infected in Europe (*n* = 184), genotype D (61%) was the main genotype followed by genotype A (28%). The majority of cases claimed to have been infected in Norway were identified with genotype D (47%) or A (33%).

**Fig. 2:**
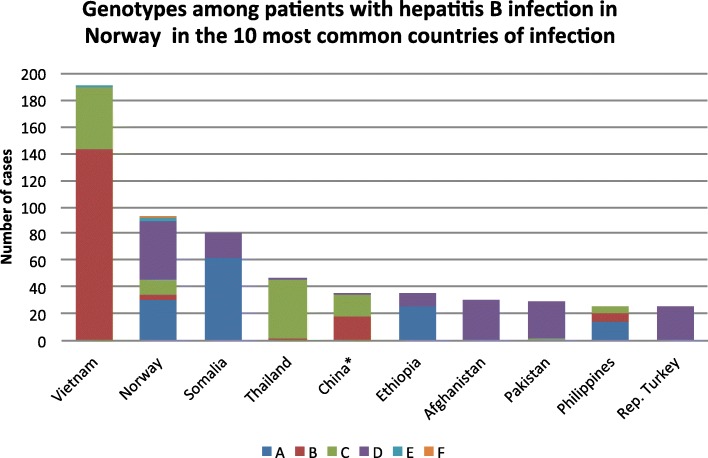
The ten most frequent countries of origin of HBV infections among patients in Norway. The distribution of genotypes is indicated on the country level. * one recombinant CD is excluded

HBV genotypes in the 10 most frequent countries indicated as origin of infection are shown in Fig. 2. These countries contribute to 65% of the total amount of cases with known origin of infection in Norway. In this regard Norway was indicated at the second most common origin of infection, representing 8% of the total cases. Immigrants from Vietnam was predominant among cases with genotypes B. Similarly, genotype C was found most frequently in immigrants from Vietnam, Thailand and China. Immigrants from Somalia dominate among patients with genotype A followed by Ethiopia and the Philippines. In contrast, genotype D was found to be more globally distributed and was also predominant among cases likely to have been infected in Norway, Afghanistan, Pakistan and Turkey. Genotype E contributed to 7% of cases and was dominated by immigrants from a variety of different countries in Africa, predominantly from West and Central Sub-Saharan Africa.

Information on likely transmission route was only available for 24% (*n* = 277) of the cases with the majority being transmitted through mother to child transmission 36% (*n* = 100), sex 33% (*n* = 91) and intravenous drug use 21% (*n* = 58). Of the cases claimed to be infected in Norway (*n* = 91) information on transmission was available for 76 cases, the majority being transmitted sexually, 33% (*n* = 30), or by IDU, 34% (*n* = 31).

**Fig. 3:**
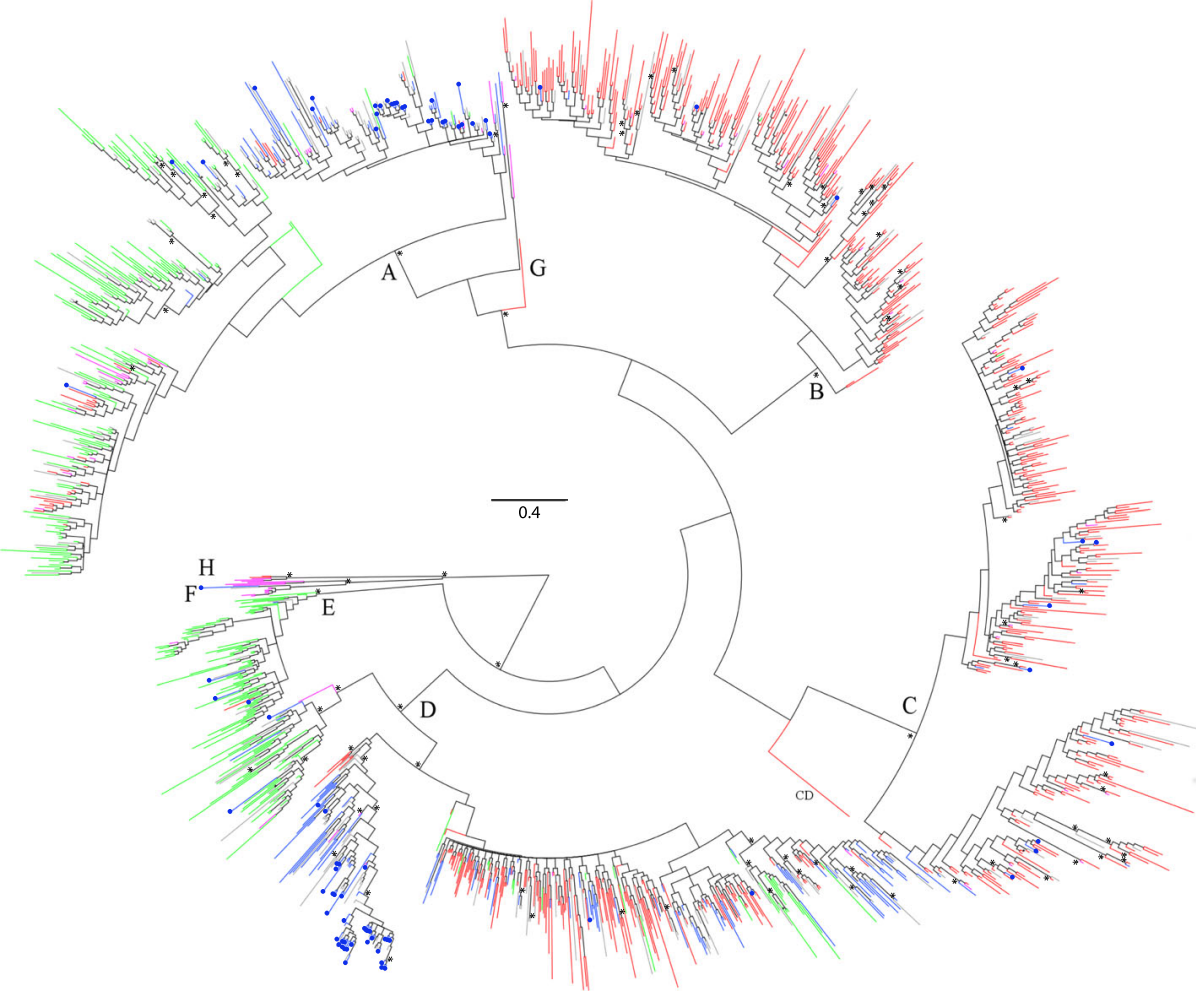
Bayesian phylogenetic tree of 1512 HBV S-gene sequences. Letters A–H designate HBV genotype. Tips are coloured according to a broadly defined geographic region where HBV was stated to have been acquired: Green = Africa; Purple = Americas; Red = Asia; Blue = Europe; Grey = Unknown geographical origin. Blue tips with blue circles are sequences of patients infected in Norway. Asterisk (*) indicates posterior probability support of ≥0.95

The phylogeny of the Norwegian HBV S-gene sequences was inferred using a Bayesian approach that included 1157 sequences collected in Norway and 355 reference sequences from NCBI GenBank. The result of the Bayesian analysis, conducted in MrBayes, was summarized as a consensus tree and is visualised in Fig. 3. There was ≥0.95 posterior probability support for the monophyletic clustering of genotypes A–F and H. However, the phylogenetic relationships between genotypes were largely unresolved. The phylogeny also showed structure with regards to geographical origin. Genotypes B, C and E were categorized by a majority of sequences originating from a single geographic region (Asia, Asia and Africa, respectively), whereas genotypes A and D included sequences from multiple geographic regions. However, within genotypes A and D, the phylogeny indicated a clustering of sequences depending on their geographical origin. That is, African, Asian and European sequences were more likely to be found in separate clusters rather than a mixture thereof. The sequences from patients stated to have become infected in Norway were found primarily within genotype A and D and were characterized by both small clusters and interspersed sequences.

## Discussion

This is the first molecular epidemiology study of HBV in Norway, where sequence information from more than 1100 diagnosed patients, tracing back to 1979, have been linked to epidemiological data on origin of infection and likely transmission route from the Norwegian surveillance system for communicable diseases (MSIS). Averaged over all years of study (1979–2011), genotype D was the most prevalent followed by relative equal distribution of genotype A, B and C. Genotype D was found to be more globally distributed and was also predominant among cases likely to have been infected in Europe (including Norway), Afghanistan, Pakistan and Turkey. Genotype A was common among African immigrants from Somalia and Ethiopia, but rarely found in Asian immigrants. The presence of other genotypes in Norway (B, C, E, H) was mostly attributed to immigration of people from South East Asia and partly from Africa. For example, genotype B is commonly found among patients from Vietnam and partly from China, where as genotype C found most frequent in immigrants from Thailand, China and Vietnam. Moreover, the sequences from people likely to have been infected in Asia (40%) and Africa (22%) mainly cluster together according to their origin of infection. This is seen in the phylogenetic tree, clearly reflecting the diverse origin of patients in Norway from other parts of the world. This also implies that HBV has been introduced to Norway on multiple occasions back in time. However, this is not surprising given that Norway since the early 1970s has experienced migration of people from many high- and intermediate endemic regions. In addition, it can be noted that for genotype A and D, the two most common genotypes in Norway, that there also has been occasional single origin epidemic outbreaks, as shown by the clustering of sequences of patients from Norway that were also infected in Norway (blue circles in Fig. 3). This most likely reflects the introduction of HBV to local units of people, such as in a community of PWID.

In our data we found that genotype D (61%) and genotype A (28%) were the dominant genotypes among persons infected in Europe (16% in total), including Norway. When exclusively looking into the cases infected in Norway, genotype D (47%) dominated whereas genotype A was found in 33% of the cases. Previous studies from Europe have similarly reported that genotype A and D dominate in Europe, but that genotype A is frequent in Northern Europe whereas genotype D is frequent in Southern Europe [26, 27]. The latter is not supported by our study, as we observe a higher prevalence of genotype D rather than genotype A among persons assumed to be infected in Norway.

The majority of the sequences from people stated to have become infected in Norway were found in clusters together or in clusters with sequences of European origin. However, some individual sequences were found to cluster with sequences from other non-European regions, indicating that these patients may have acquired their infection outside Europe or in Norway via a person previously infected outside Europe. We believe that this is partly due to the challenge of accurate reporting of origin of infection to MSIS due to the high number of cases among migrants that may not know their infections status before symptoms in adulthood and the difficulty to get accurate and verifiable information from these cases. It is important to note that we have not been able to distinguish between native Norwegians and migrants in this study. As such, the relatively high prevalence of genotype D in Norway may therefore reflect the origin of the migrant population from regions where genotype D is more prevalent. Furthermore, the transmission route is not known in the majority (83%) of cases according to MSIS [8]. Therefore, likely route of transmission could not be presented in detail.

The interpretation of the molecular epidemiology and the transmission dynamics of HBV in our study was limited by sequence length and epidemiological data. Although the phylogenetic tree accurately reconstructs the various genotypes into distinct clades, complete genome sequence information would be needed to make detailed epidemiological inferences as well as to identify sub-genotypes and distinct clusters [11]. Sub-genotyping, that may have given a better understanding of the different genotype pattern observed in Norway compared to other Northern European countries in particular, was therefore not performed as part of the study. Norway was reported as the second most frequent country of infection (Fig. 2), representing 8% of the total study population, but this is not supported by the phylogenetic analysis in several cases. Lack and uncertainty of the epidemiological data make the interpretation of the transmission dynamics difficult. Further, the ethical approval for combing epidemiological data from MSIS with HBV sequence data was limited to samples collected and analysed until 2011. Analysed sequence beyond this period was therefore excluded. Regardless of these limitations, this is the first molecular epidemiological study on HBV in Norway with more than 1100 sequences collected from all over Norway as genotyping is only performed at the national reference laboratory for hepatitis in Norway at the Norwegian Institute of Public Health as part of patient management. Given that the genotype distribution and that the immigrant population has been relatively stable, the data presented herein for the years 1979–2011 may inform current public health and treatment strategies. This is important, as highlighted previously, as the diversity of genotypes considerably differ with respect to geographical distribution, transmission routes, disease progression, responses to antiviral therapy and clinical outcomes [14, 28–30]. HBV genotyping is therefore relevant in diagnostics both from a clinical and epidemiological perspective, and to better understand the source of HBV in Norway and other countries.

## Conclusions

In this study we observed a great mix of genotypes among patients in Norway. HBV infections in Norway are mainly driven by an influx of people with chronic infection who acquired their HBV infection prior to arrival in Norway. The overall genotype distributions in Norway therefore mirror that of the origin of the migrant population to a large extent. Given a population with a great mixture of genotypes, genotyping is important to identify chronic HBV infected at higher risk of liver disease progression enabling optimisation of management and antiviral therapy for these patients.

## Additional file

Additional file 1: **Table S1.** List of 355 publically available sequences, with meta-information, included in the phylogenetic analysis. (XLSX 20 kb)

## Abbreviations

GTR: General time reversible model
HBsAg: Hepatitis B surface antigen
HBV: Hepatitis B virus
IDU: Intravenous drug use
MCMC: Markov chain Monte Carlo
MSIS: Norwegian surveillance system for communicable diseases
MSM: Men who have sex with men
MTCT: Mother to child transmission
PWID: People who inject drugs
REC: Regional committee for medical and health research ethics
